# Effects of monochromatic red and blue light on photosynthetic efficiency and nutritional quality in Chinese kale microgreens

**DOI:** 10.3389/fpls.2026.1781319

**Published:** 2026-04-17

**Authors:** Honghua Yu, Xiaodong Chen, Zhanfeng Yang, Jiaxuan Chen, Chenhao Guan, Hao Weng, Rongfang Guo

**Affiliations:** 1 Joint Fujian Agriculture and Forestry University (FAFU)-Dalhousie Lab, College of Horticulture, Fujian Agriculture and Forestry University, Fuzhou, China; 2 Key Laboratory of Ministry of Education for Genetics, Breeding and Multiple Utilization of Crops, Fujian Agriculture and Forestry University, Fuzhou, China

**Keywords:** blue light, Chinese kale, chlorophyll, GLK2, red light

## Abstract

Microgreens, a type of rapidly grown and consumed vegetable, are rich in secondary metabolites. Chlorophyll serves as a crucial component contributing to their visual quality and is also recognized as a key nutritional constituent in Chinese kale microgreens. This study employed an integrated physiology-transcriptomics approach to investigate how preharvest light quality modulates metabolic pathways in microgreens. Our findings demonstrate that blue light (450 nm) preferentially enhances chlorophyll b content (1.72-fold) while improving photosynthetic efficiency through upregulation of photosystem I/II subunits and light-harvesting complex genes. This spectral treatment significantly promoted chlorophyll biosynthesis, increased soluble sugar accumulation by 154.85%, and activated cryptochrome-mediated signaling pathways. Conversely, red light (660 nm) induced morphological expansion but suppressed chlorophyll accumulation through phytochrome signaling-mediated repression of key biosynthetic enzymes (POR/CAO) and Mg-chelatase components. Transcriptomic analysis further revealed blue light’s stabilization of chlorophyll biosynthetic enzymes and suppression of degradation pathways via GLK2. These findings provide molecular insights into spectral regulation of plant metabolism and propose practical LED lighting strategies for optimizing nutritional quality and visual characteristics of microgreens in controlled environment agriculture.

## Highlight

1. Increased content of chlorophyll under blue-light conditions.
2. *GLK2*’s expression was enhanced by blue-light treatment.
3. Regulation of chlorophyll biosynthesis by GLK2 in microgreens.

## 1 Introduction

The spectral composition of light significantly influences the food quality attributes of microgreens, particularly through modulation of metabolism in microgreens. Chlorophyll derivatives serve dual roles as natural food colorants and bioactive compounds, with their stability and bioactivity significantly influenced by preharvest light conditions (Toscano et al., 2021). The magnesium-centered tetrapyrrole structure of chlorophyll, particularly its oxidative stability during postharvest handling, directly impacts both the visual appeal and functional properties of microgreens as fresh-cut products. The stability and bioavailability of chlorophyll are critically affected by preharvest light conditions, making spectral tuning a powerful tool for quality optimization.

Recent advances in photobiology have revealed that blue light (400–500 nm) and red light (600–700 nm) differentially regulate both primary and secondary metabolic pathways (Wang et al., 2016). Similar spectral effects on morphological and physiological traits have been observed in woody species such as the GF677 rootstock, where different blue-red LED intensities modulated *in vitro* growth and development (Sarropoulou et al., 2024). Blue light specifically enhances chlorophyll b synthesis through post-translational activation of chlorophyll a oxygenase (CAO) (Biswal et al., 2012), while simultaneously increasing glucosinolate content (Zhang et al., 2020b). In contrast, red light promotes morphological growth but reduces chlorophyll accumulation through phytochrome-mediated suppression of key biosynthetic enzymes (Luimstra et al., 2020). These wavelength-specific effects create opportunities for targeted nutritional enhancement, particularly important given consumer preferences for intensely green microgreens (Kyriacou et al., 2019).

At the molecular level, light-regulated chlorophyll metabolism involves multi-layer controls that ultimately determine food quality parameters. The blue light-induced stabilization of chlorophyll biosynthetic enzymes (Matters and Beale, 1995) and red light-mediated suppression of degradation pathways (Lu et al., 2015) create complementary approaches for optimizing both chlorophyll content and stability in postharvest handling.

Chinese kale microgreens represent an ideal model system for studying photosynthesis-metabolism coupling due to their rapid growth cycle, high nutrient density, and sensitivity to light quality (Chen et al., 2021). An integrated food chemistry approach by combining physiological measurements and transcriptomic analysis is employed in this study to investigate how chlorophyll metabolism and overall nutritional quality in Chinese kale microgreens are influenced by monochromatic light treatments. Our findings provide both fundamental insights into light-mediated chlorophyll metabolism and practical lighting strategies for controlled environment agriculture, addressing the growing industry demand for nutritionally optimized, visually appealing microgreen products.

## 2 Materials and methods

### 2.1 Plant material and growth conditions

Chinese kale (*Brassica oleracea* var. *alboglabra*) cv. ‘HuangHua’ was obtained from Gaoda Seedling Co., Ltd. (Fuzhou, China). Seeds were surface-sterilized with 2% (20 g/L) sodium hypochlorite for 10 min, rinsed thoroughly with deionized water, and germinated on perlite-filled glass trays (25 × 15 × 5 cm) in controlled-environment chambers (PGX-450C, Ningbo Jiangnan Instrument, China) at Fujian Agriculture and Forestry University. Growth conditions were maintained at 25 °C with 65% relative humidity. After 24 h of dark germination, non-germinated seeds were removed. Microgreens were harvested at 3 days and 6 days post-illumination (DPI), respectively. At 3 and 6 days post-illumination, the plants had fully expanded cotyledons and were actively photosynthesizing under the provided light conditions, corresponding to the microgreen stage of development. For each biological replicate (n = 5), cotyledons and hypocotyls were separately collected from at least 15 seedlings. For morphometric analysis, fresh samples were immediately photographed under standardized conditions (Canon EOS 90D with 18–55 mm lens, white balance calibrated). For physiological assays: tissues were flash-frozen in liquid N_2_ and stored at -80 °C until analysis.

### 2.2 Light treatments

Two spectral regimes were established using LED panels (KL5T8A-120W, Huizhou Kedao Technology, China): monochromatic red light (R, peak λ = 660 ± 5 nm); monochromatic blue light (B, peak λ = 450 ± 5 nm). All treatments maintained a photosynthetic photon flux density (PPFD) of 150 μmol·m^-^²·s^-^¹ at canopy level, measured by quantum sensor (LI-250A, LI-COR Biosciences, USA). Spectral distributions were verified via a spectrometer (AvaSpec-2048, Avantes, Netherlands).

### 2.3 Chlorophyll fluorescence analysis

Chlorophyll fluorescence parameters were measured using an IMAGING-PAM M-series modulated fluorescence system (Heinz Walz GmbH, Germany). Cotyledons from each treatment group (n=4 biological replicates) were dark-adapted for 30 min before measurement. The following parameters were recorded: minimal fluorescence (F_0_) under weak measuring light; Maximal fluorescence (F_m_) during saturating pulse (3000 μmol·m^-^²·s^-^¹, 800 ms); Maximum quantum yield of PSII (F_v_/F_m_ = (F_m_ - F_0_)/F_m_); Effective quantum yield of PSII (ΦPSII) under actinic light; Non-photochemical quenching (NPQ); Photochemical quenching coefficient (qP); Measurements were performed at 25 °C with 60% relative humidity. Data were analyzed using ImagingWin software (v2.41, Walz).

### 2.4 Photosynthetic pigment quantification

Pigments were extracted from 100 mg fresh tissue (cotyledons/hypocotyls) with 1 mL 100% ethanol (HPLC grade, Sigma-Aldrich) in darkness at 4 °C for 24 h. Absorbance was measured at 470, 645, and 663 nm using a UV-Vis spectrophotometer (UV-2600, Shimadzu, Japan). Concentrations were calculated as:


$$Chl\;a\;\left(\mu g/mL\right)\;=\;13.95A_{663}\;-\;6.88A_{645};$$

$$Chl\;b\;\left(\mu g/mL\right)\;=\;24.96A_{645}\;-\;7.32A_{663};$$

Total carotenoids (μg/mL) = (1000A_470_ - 2.05[Chl a] - 114[Chl b])/245.

Results were normalized to fresh weight (FW) and dry weight (DW) after lyophilization.

### 2.5 Soluble sugar determination

Soluble sugars were extracted from 0.1 g tissue in 1 mL distilled water, homogenized (Polytron PT 1200E, Kinematica, Switzerland), and heated at 95 °C for 10 min. After centrifugation (8000 × g, 10 min, 25 °C), supernatants were diluted to 10 mL. Anthrone reagent was prepared by dissolving 0.2 g anthrone (Sigma-Aldrich) in 100 mL 80% sulfuric acid. Reaction mixtures contained: Blank: 1000 μL H_2_SO_4_ + 400 μL H_2_O + 100 μL reagent; Sample: 1000 μL H_2_SO_4_ + 200 μL extract + 200 μL H_2_O + 100 μL reagent. After 10 min at 95 °C, absorbance at 620 nm was measured. Sugar content was calculated using:

Soluble sugar (mg/g FW) = 1.17 × (ΔA_620_ + 0.07)/sample weight (g).

### 2.6 Soluble protein assay

Proteins were extracted from 0.1 g tissue in 5 mL ice-cold PBS (pH 7.4) followed by centrifugation (8000 × g, 10 min, 4 °C). The BCA assay (Pierce™ Kit, Thermo Scientific) was performed by mixing 20 μL H_2_O, 20 μL BSA (0.5 mg/mL), and 20 μL supernatant with 1 mL working reagent, respectively, as blank, standard, and sample. After incubation (60 °C, 30 min), absorbance at 562 nm was measured. Protein concentration was calculated as:

Protein (mg/g FW) = 0.5 × (A_562__sample - A_562__blank)/(A_562__standard - A_562__blank)/sample weight (g).

### 2.7 RNA extraction and library preparation

For transcriptomic analysis, total RNA was extracted from whole seedlings (including both cotyledons and hypocotyls) of Chinese kale microgreens using the RNAiso Plus kit (Takara, Cat. No.9109). Three biological replicates were prepared for each treatment group (HHR: 100% red light; HHB: 100% blue light), with each replicate consisting of pooled tissue from at least 10 seedlings. RNA concentration and purity were assessed using a NanoDrop 1000 spectrophotometer (Thermo Fisher Scientific, USA) and Qubit 2.0 Fluorometer (Life Technologies, USA). RNA integrity was verified by agarose gel electrophoresis (RIN > 8.0). Poly(A)+ mRNA was enriched using Oligo (dT) magnetic beads, followed by rRNA depletion using a Ribo-Zero rRNA Removal Kit (Illumina, USA). The purified mRNA was fragmented and reverse-transcribed into double-stranded cDNA using the NEBNext Ultra II RNA Library Prep Kit (NEB, Cat. No. E7770 USA). Sequencing libraries were constructed through end repair, A-tailing, adapter ligation, and PCR amplification. Library quality was assessed using an Agilent 2100 Bioanalyzer (Agilent Technologies, USA) and quantified via qPCR (ABI StepOnePlus, Thermo Fisher Scientific).

### 2.8 RNA sequencing and data processing

Paired-end sequencing (150 bp) was performed on the BGISEQ-500 platform (BGI, Shenzhen, China). Raw reads were filtered to remove low-quality sequences (Q< 20), adapter contamination, and reads with >10% N bases using SOAPnuke (v2.0). Clean reads were aligned to the Brassica oleracea reference genome (Ensembl Plants) using HISAT2 (v2.2.1). Gene expression levels were quantified as FPKM (Fragments Per Kilobase per Million mapped reads) using RSEM (v1.3.3).

Differentially expressed genes (DEGs) were identified using the DEGseq R package (v1.44.0) with thresholds of |log_2_ (fold change) | > 2 and adjusted p-value (Q-value) ≤ 0.001. Gene ontology (GO) enrichment and Kyoto Encyclopedia of Genes and Genomes (KEGG) pathway analyses were performed using Blast2GO (v5.2) and KOBAS (v3.0), respectively. Arabidopsis thaliana orthologs were used for functional annotation. Raw sequencing data were deposited in the NCBI SRA database (Accession: PRJNA649862).

### 2.9 Statistical analysis

Statistical analyses were performed using SPSS Statistics 26 (IBM, USA) and GraphPad Prism 8 (GraphPad Software, USA). Data normality was verified using the Shapiro-Wilk test (α = 0.05), and homogeneity of variance was confirmed by Levene’s test (p > 0.05). One-way analysis of variance (ANOVA) was applied to assess differences among treatment groups, followed by Duncan’s multiple range test at 95% confidence level to determine significant differences between means, which are indicated by superscript letters in figures. All data are presented as mean ± standard deviation (SD) from at least three biological replicates. Heatmaps were generated using TBtools (v2.025) with hierarchical clustering based on Euclidean distance and complete linkage to visualize expression patterns of differentially expressed genes and metabolite profiles.

## 3 Results

### 3.1 Effects of light quality on chlorophyll fluorescence characteristics in Chinese kale microgreens

**Figure 1:**
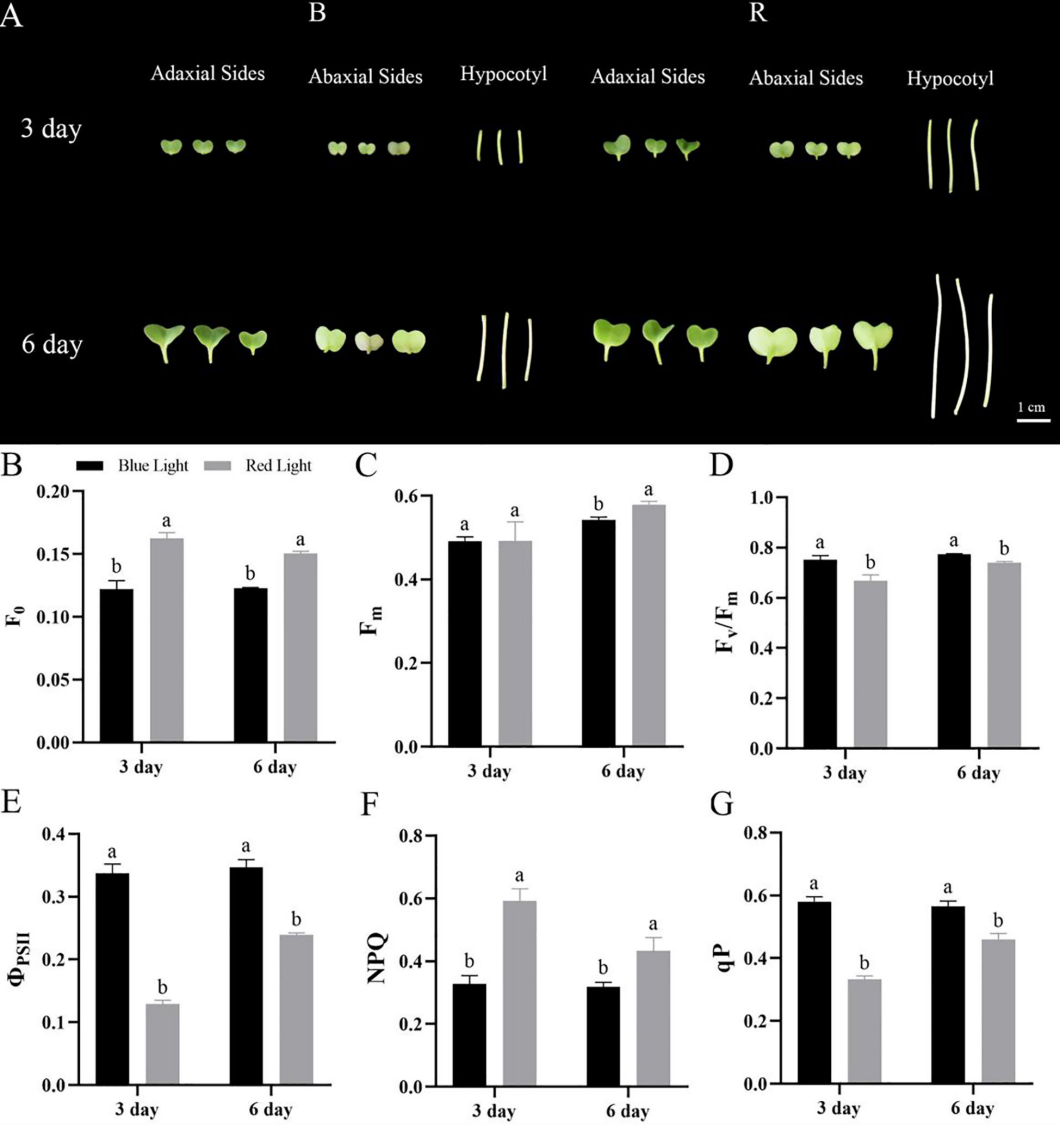
Effect of different culture time and light quality on the morphology of cotyledon and hypocotyl, as well as chlorophyll fluorescence parameters of Chinese kale microgreens. **(A)** Morphology of Chinese kales adaxial cotyledon, abaxial cotyledon, and hypocotyl under blue and red light conditions. **(B)** Initial fluorescence (F0). **(C)** Maximal fluorescence (Fm). **(D)** Maximum quantum yield of primary photochemistry (Fv/Fm). **(E)** Actual photosynthetic efficiency (ΦPSII). **(F)** Photochemical quenching (NPQ) and **(G)** photochemical quenching (qP). Data are presented as mean ± SD (n = 5 biological replicates). Different letters indicate significant differences between treatments (one-way ANOVA followed by Duncan’s multiple range test, p< 0.05).

Chinese kale microgreens exhibited distinct morphological responses to different light quality treatments (**Figure 1A**). Phenotypic analysis revealed that red light promoted the development of larger cotyledons, elongated petioles, and increased hypocotyl elongation compared to blue light treatments. Chlorophyll fluorescence parameters demonstrated significant light quality-dependent variations (**Figures 1B–D**). Blue light-treated microgreens showed reduced initial fluorescence (F0) and maximal fluorescence (Fm), but enhanced maximum quantum yield (Fv/Fm) of PSII photochemistry relative to red light. The lower F0 under blue light suggests reduced excitation energy loss in PSII reaction centers under dark-adapted conditions, likely reflecting fewer inactive PSII centers or more efficient energy transfer to the reaction centers. The reduced Fm, combined with higher Fv/Fm, indicates that a greater proportion of PSII reaction centers are photochemically competent and capable of efficient charge separation. In contrast, red light exposure resulted in elevated F0, suppressed Fm, and significantly lower Fv/Fm values, suggesting an increased proportion of inactive PSII centers or accumulation of damaged reaction centers, indicative of photoinhibition or stress conditions.

Analysis of light-adapted fluorescence parameters revealed that blue light significantly enhanced both the effective quantum yield of PSII and photochemical quenching coefficient (qP) compared to red light treatments at all growth stages (**Figures 1E–G**). The 154.85% higher ΦPSII under blue light indicates superior photosynthetic energy conversion efficiency. The reduced qP under red light implies a higher proportion of closed PSII reaction centers, suggesting impaired electron transport chain activity downstream of PSII. Non-photochemical quenching (NPQ) showed no significant differences between spectral treatments, suggesting comparable thermal dissipation capacity. However, the 14.77% reduction in qP under red light implies impaired electron transport chain activity and reduced photochemical conversion efficiency relative to blue light conditions. These findings collectively demonstrate that blue light optimizes photosynthetic performance in Chinese kale microgreens, while red light appears less favorable for photochemical processes despite promoting morphological expansion.

### 3.2 Physiological responses of Chinese kale microgreens to red and blue light treatments

**Figure 2:**
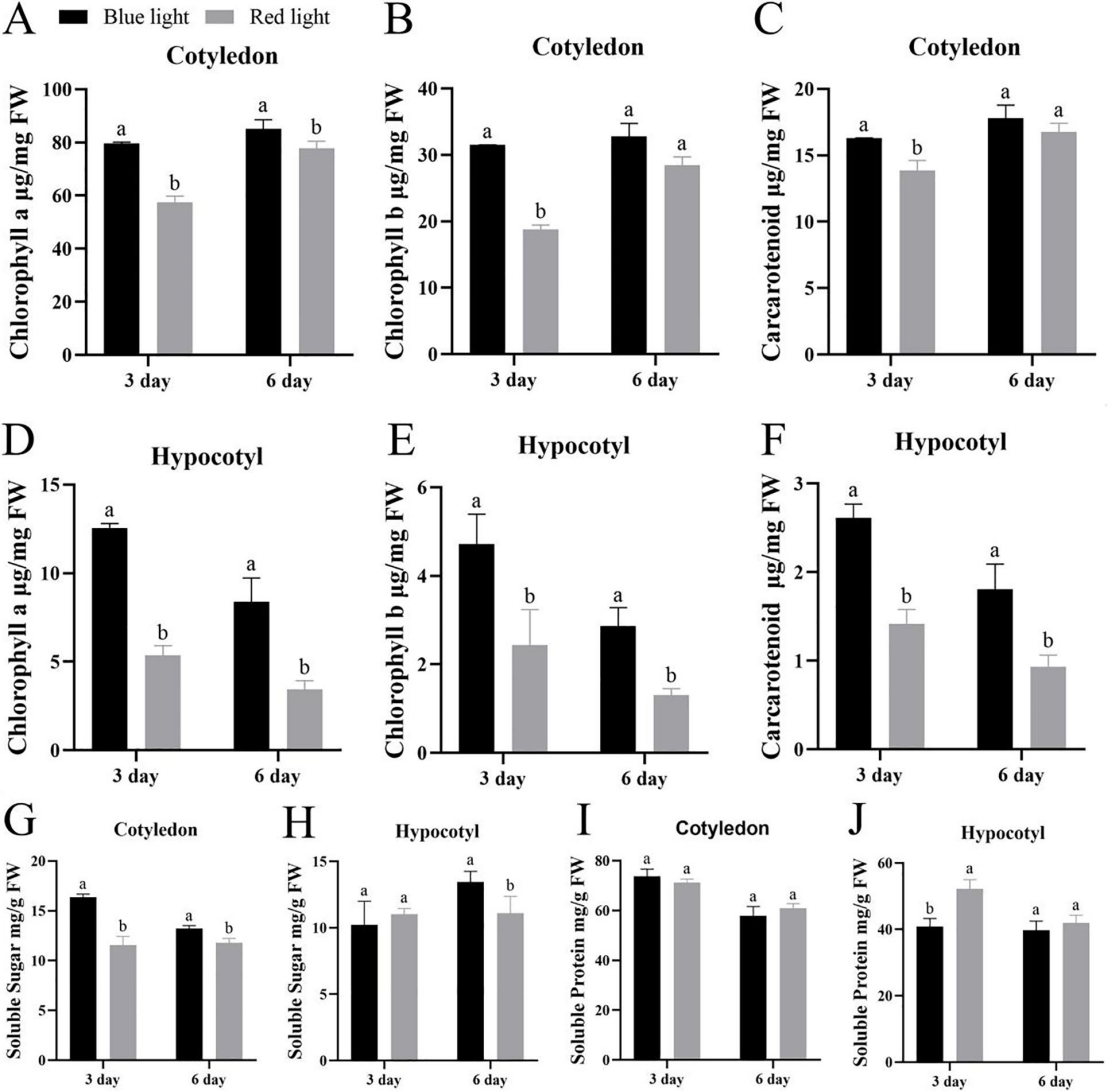
The photosynthetic pigment content, the soluble sugar, and the soluble protein content of different parts of Chinese kale microgreens under different culture time and light quality treatments. **(A)** Chlorophyll a content in the cotyledons of Chinese kale microgreens. **(B)** Chlorophyll b content in the cotyledons of Chinese kale microgreens. **(C)** Carotenoid content in the cotyledons of Chinese kale microgreens. **(D)** Chlorophyll a content in the hypocotyls of Chinese kale microgreens. **(E)** Chlorophyll b content in the hypocotyls of Chinese kale microgreens. **(F)** Carotenoid content in the hypocotyls of Chinese kale microgreens. **(G)** Respectively represent the soluble sugar content in cotyledons. **(H)** Respectively represent the soluble protein content in hypocotyls. **(I)** Respectively represent the soluble sugar content in cotyledons. **(J)** Respectively represent the soluble protein content in hypocotyls. Data are presented as mean ± SD (n = 5 biological replicates). Different letters indicate significant differences between treatments (one-way ANOVA followed by Duncan’s multiple range test, p< 0.05).

The photosynthetic pigment composition of Chinese kale microgreens exhibited significant tissue-specific and light quality-dependent variations (**Figures 2A–F**). Cotyledons consistently maintained higher chlorophyll a (Chl a), chlorophyll b (Chl b), and carotenoid contents compared to hypocotyls across all treatments. In cotyledons, pigment accumulation showed a developmental progression, with blue light inducing the most substantial pigment enrichment at day 3, representing a 38.84%, 67.87% and 17.7% increase over red light controls, respectively. Red light treatment resulted in significantly lower pigment levels at this stage, with carotenoid content being particularly reduced. While light quality effects on cotyledon pigments diminished by day 6, hypocotyls displayed a contrasting pattern with progressive pigment reduction under red light. The carotenoid/chlorophyll ratio, an indicator of photoprotective capacity, was consistently lowest in red-light-treated hypocotyls, suggesting compromised antioxidant protection and light energy dissipation mechanisms.

Carbohydrate and protein metabolism showed complex spatiotemporal regulation (**Figure 2**). Soluble sugar accumulation patterns differed markedly between tissues (**Figures 2G, H**). Cotyledons exhibited stage-dependent variations, with blue light promoting maximal sugar content by day 3, while red light induced lower accumulation than blue light in cotyledons. Hypocotyls demonstrated stronger light quality discrimination, with blue light maintaining superior sugar levels during early development and significantly surpassing red light at day 6. Protein content analysis revealed minimal differences between red and blue light treatments in both tissues after 6 days (**Figures 2I, J**).

### 3.3 Transcriptome analysis of Chinese kale Microgreens under red or blue light conditions

**Figure 3:**
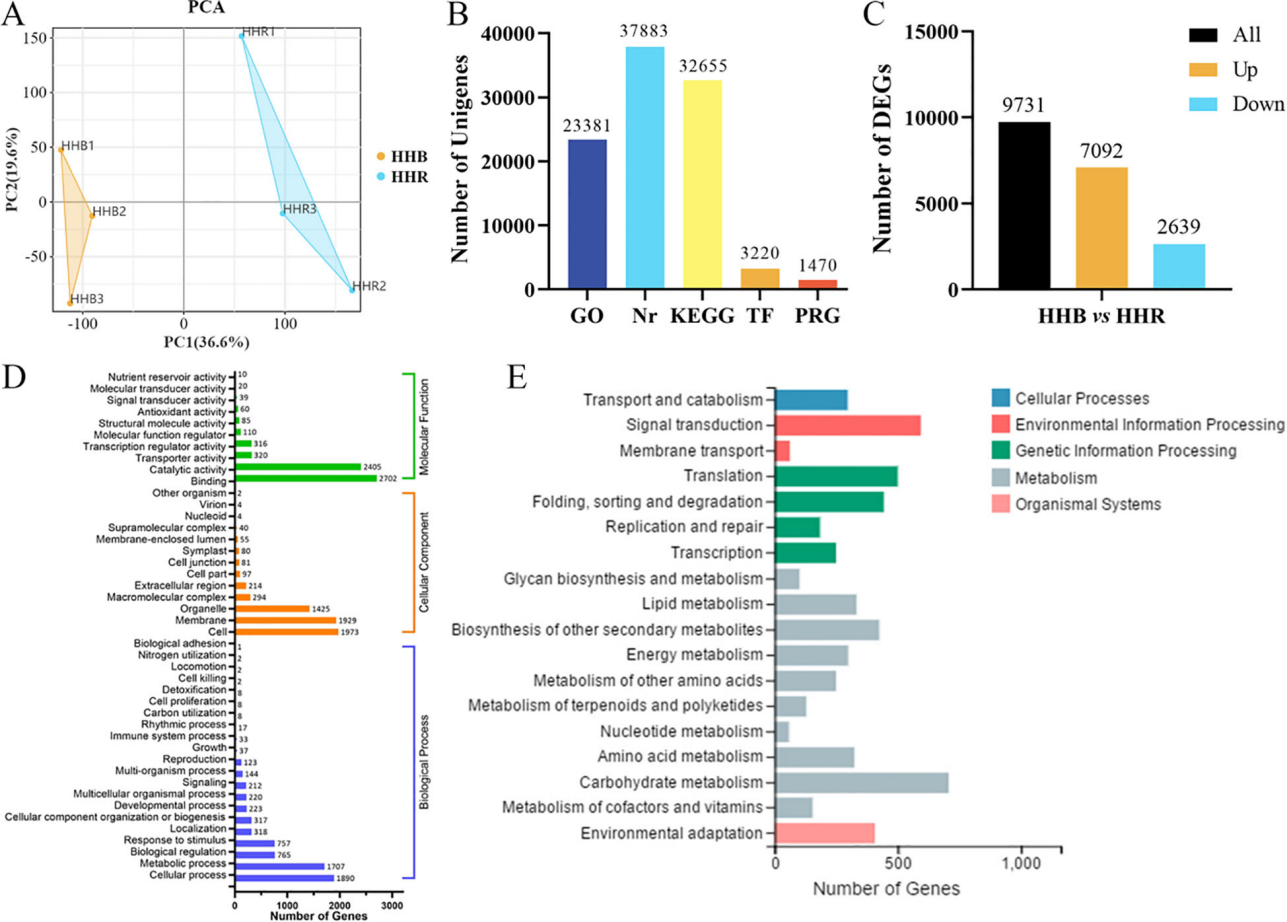
Illumina sequencing and transcriptomes under red (HHR) and blue light (HHB). **(A)** Principal component analysis (PCA) between two treatments and three repetitions. **(B)** The annotation of unigenes is based on the listed databases. **(C)** Number of DEGs in comparison of the two treatments. **(D)** GO analysis of DEGs between HHR and HHB groups in three main categories. **(E)** Classification of enriched KEGG terms, including pathways related to cellular processes, environmental information processing, genetic information processing, metabolism, and organismal systems. Data are based on three biological replicates per treatment.

RNA-seq analysis was performed on 6-day-old microgreens grown under red (R) and blue (B) light conditions using the Illumina HiSeq 4000 platform, generating an average of 45 million clean reads per sample (Q30 > 90%). Principal component analysis (PCA) revealed clear separation between light treatments along PC1 (72.3% variance), while biological replicates clustered tightly within groups (**Figure 3A**), confirming experimental reproducibility.

Assembly of 246 million paired-end reads produced 41, 538 non-redundant transcripts (N50 = 2, 154 bp), including 3, 414 novel genes not present in *Brassica* reference genomes. Functional annotation success rates were: 38.42% in the Nr database, 33.12% in KEGG, 23.71% in GO, with 3.26% and 1.49% representing transcription factors and plant resistance genes, respectively (**Figure 3B**). Comparative analysis identified 2, 843 differentially expressed genes (DEGs) (|log2FC| > 2, FDR< 0.01), with 1, 572 upregulated and 1, 271 downregulated in B vs R (**Figure 3C**). Gene Ontology analysis revealed significant enrichment in binding (1, 842 genes) and catalytic activity (1, 576 genes) for molecular functions, cellular components (cell, membrane, organelle), and metabolic processes (2, 287 genes) (**Figure 3D**). KEGG pathway analysis identified photosynthesis as the most enriched pathway, followed by carbohydrate metabolism and signal transduction (**Figure 3E**). These results demonstrate light quality’s predominant effects on photosynthetic and metabolic pathways in Chinese kale microgreens.

### 3.4 Transcriptional regulation of photosynthesis and chlorophyll biosynthesis pathways in response to light quality

**Figure 4:**
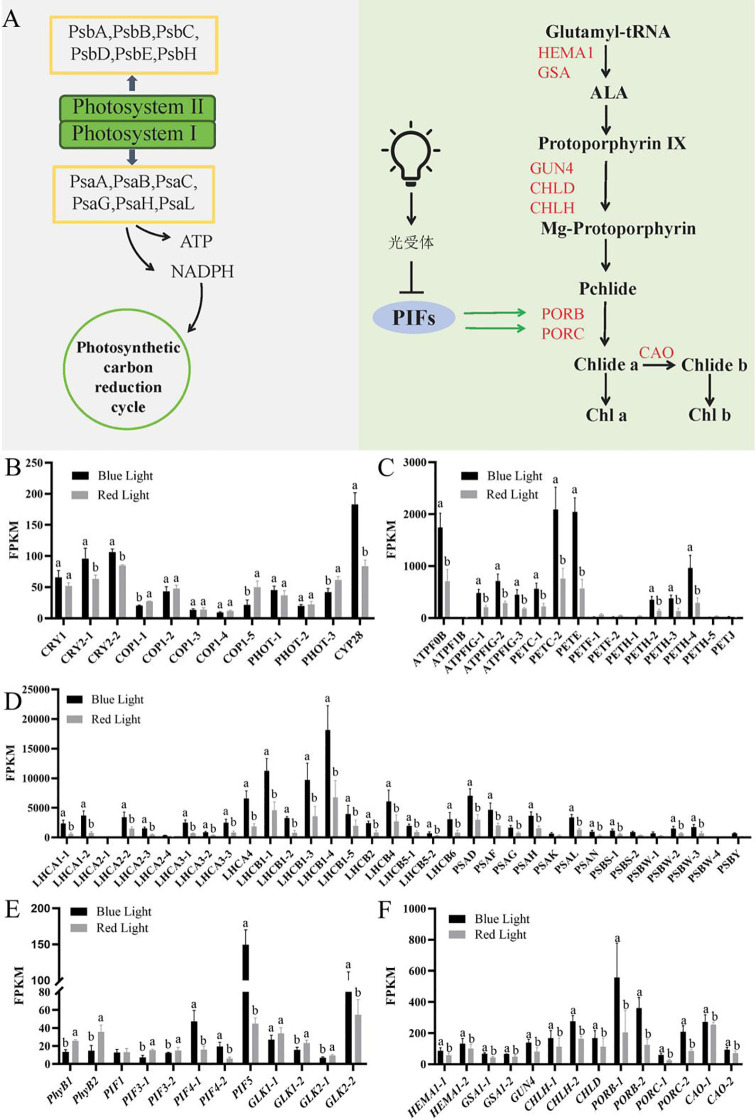
Photosynthetic regulation of the chlorophyll synthesis network and related gene expression. **(A)** A regulatory network of Photosynthetic and chlorophyll biosynthesis by light. **(B)** FPKM of genes related to the blue light photoreceptors. **(C, D)** FPKM of genes related to the photosynthesis pathways. **(E)** FPKM of genes related to the chlorophyll regulatory factors. **(F)** FPKM of genes related to the chlorophyll synthesis pathways. Data are based on three biological replicates per treatment. Expression values are shown as FPKM (Fragments Per Kilobase per Million mapped reads). Asterisks indicate significant differences between treatments (|log_2_FC| > 2, FDR < 0.01). CRY, Cryptochrome; COP1, Constitutive Photomorphogenic 1; PHOT, Phototropin; ATPF, ATP synthase components; PET, Ferredoxin-NADP reductase genes; LHC, Light-harvesting Chlorophyll a/b-binding Protein; PSA, Photosystem I subunits; PSB, Photosystem II subunits; phyB, Phytochrome B; PIF, Phytochrome-Interacting Factors; GLK, GOLDEN2 – LIKE; HEMA, Glutamyl; tRNA, Reductase; GSA, Glutamate - 1 – Semialdehyde; GUN, Genomes Uncoupled; CHL, Light - independent protochlorophyllide reductase iron-sulfur ATP - binding protein; POR – NADPH, Cytochrome P450 Oxidoreductase; CAO, Chlorophyllide a Oxygenase gene.

Transcriptome analysis revealed distinct light-quality-dependent regulation of photosynthesis-related genes in Chinese kale microgreens (**Figure 4A**). Among 30 photosynthesis-related genes annotated in KEGG pathways, 76.67% (23/30) demonstrated elevated expression in HHB, including photosystem I subunits (*PSA* genes, 1.92-2.83- fold increase), photosystem II subunits (*PSB* genes, 2.08-2.45- fold increase, except *PSB27-1*), ATP synthase components (*ATPF* genes, 2.31-2.76- fold increase), and ferredoxin-NADP reductase genes (*PET* family, 1.78-3.26- fold increase) (**Figures 4C, D**). Differential expression analysis identified 20 *light-harvesting complex* (*LHC*) genes, comprising 10 *LHCA* and 10 *LHCB* family members, all of which showed significantly higher expression (2.05 - to 4.92-fold) under blue light (HHB) compared to red light (HHR) treatment (**Figure 4D**). Notably, *LHCB1–4* exhibited the most pronounced upregulation (2.69 -fold) under blue light conditions.

The chlorophyll biosynthesis pathway analysis identified three critical regulatory nodes affected by light quality (**Figure 4A**). First, genes involved in the rate-limiting step of ALA synthesis, particularly *HEMA1-2* (1.48-fold) and *GSA1* (1.52-fold), showed significantly higher expression under blue light. Second, at the chlorophyll-hemoglobin branching point, key components of Mg-chelatase (*CHLD* and *CHLH*) exhibited 1.57-fold greater expression in HHB. Third, light-dependent conversion steps mediated by protochlorophyllide oxidoreductases (*POR*) and chlorophyll a oxygenase (*CAO*) were significantly suppressed (2.02-fold) under red light conditions (**Figure 4F**). Fourth, the expression levels of key genes (*CRY* and *CYP28*) that regulate plant photomorphology were 1.25-fold and 2.05-fold higher in HHB, respectively (**Figure 4B**).

Phytochrome-PIF signaling analysis revealed that *PhyB* expression increased 2.18-fold under red light, while downstream *PIF4/5* factors showed inverse regulation. Consequently, the expression of *PORB*/*PORC* and *CAO* genes, which are essential for chlorophyllide conversion, was significantly repressed in HHR (**Figure 4E**). Moreover, the expression of *GOLDEN2 - LIKE* (*GLK2*) was upregulated under blue light conditions but downregulated under red light. These transcriptional changes provide a molecular explanation for the observed chlorophyll deficiency under red light, with coordinated downregulation of multiple biosynthetic steps, including ALA production, Mg-chelatase activity, and light-dependent conversion reactions. The data collectively demonstrate that blue light preferentially activates both light-harvesting complex expression and chlorophyll biosynthesis, while red light triggers a transcriptional program leading to chlorophyll deficiency through modulation of the PhyB signaling cascade and subsequent repression of key biosynthetic enzymes.

## 4 Discussion

### 4.1 Light quality effects on nutritional composition and photosynthetic performance

Our integrated analysis demonstrates that blue light specifically enhances the accumulation of chlorophyll a in Chinese kale microgreens compared to red light. Notably, the superior photosynthetic pigment accumulation under blue light correlates with enhanced nutritional quality, supporting contemporary research on the health benefits of chlorophyll-rich microgreens (Lee et al., 2017). The photosynthetic efficiency parameters are directly supported by chlorophyll fluorescence lifetime imaging in Brassica microgreens (Gorbe and Calatayud, 2012), which showed comparable quantum yield differences. The enhanced photosynthetic performance and sugar accumulation observed under blue light, particularly in hypocotyls, underscore the wavelength-specific regulation of plant photophysiology and carbon metabolism. This is consistent with observations in cherry rootstock, where specific blue-red LED spectrum significantly improved photosystem II functionality and increased chlorophyll and carotenoid content during *in vitro* proliferation during *in vitro* proliferation (Sarropoulou et al., 2022). Recent studies have reinforced that blue light (400–500 nm) not only drives chlorophyll and carotenoid biosynthesis but also fine-tunes photosystem stoichiometry, favoring a more balanced excitation of PSI and PSII compared to red light (Banaś et al., 2024). This spectral optimization minimizes excess excitation pressure, explaining the observed reduction in NPQ—a finding consistent with (Chen et al., 2023), who demonstrated that blue light increases the net photosynthesis rate in grapevine plantlets.

The observed tissue-specific photoresponses in our study, particularly the more pronounced metabolic alterations in hypocotyls compared to cotyledons, are consistent with recent advances in spatially resolved light signaling mechanisms. A growing body of evidence has established cryptochrome CRY1 as the primary photoreceptor governing hypocotyl development through multiple molecular pathways. Notably, CRY1-mediated suppression of both the COP1/SPA ubiquitin ligase complex and PIF4 transcription factors constitutes a central regulatory node for hypocotyl elongation control (Boccaccini et al., 2020; Yang et al., 2023). Complementing these findings, Landi et al. (2021) identified that the efficient absorption of blue light more effectively promotes the stabilization of light-harvesting complexes, which is essential for maintaining photosynthetic functionality during photomorphogenesis. Collectively, these studies provide a comprehensive mechanistic framework that positions CRY1 as the master regulator coordinating multiple aspects of hypocotyl photomorphogenesis, from cellular elongation to photosynthetic apparatus assembly.

Microgreens grown under blue light exhibit significantly increased soluble sugar content in their hypocotyls. This blue light-induced sugar accumulation has direct implications for crop quality and nutritional value. For instance, supplemental blue lighting has been shown to enhance sugar levels in bayberry fruit (Shi et al., 2016), tomato seedlings (Gao et al., 2025), and cucumber (Palma et al., 2022), thereby improving their edible quality. Furthermore, blue light exposure promotes the accumulation of pigments and phenolic compounds in microgreens. This demonstrates that blue light can modulate carbon partitioning pathways to favor the synthesis of high-value phytochemicals (Samuolienė et al., 2017; Zhang et al., 2020a). These findings align with our observations and further support that spectral tuning can effectively enhance the nutritional and functional properties of edible plants.”

The lower NPQ under blue light challenges the traditional view that high-energy wavelengths inevitably induce photoprotective dissipation. Instead, recent models propose that blue light perception contributes to photoprotection via photoreceptor cryptochromes and the faster disassembly of prolamellar (Banaś et al., 2024; Morales et al., 2025). This aligns with our results, suggesting that blue light’s efficiency lies not only in its absorption by chlorophyll but also in its ability to activate energy-balancing mechanisms.

The enhanced photosynthetic performance under blue light, characterized by higher Fv/Fm and ΦPSII (**Figures 1D, E**), is supported at the molecular level by the upregulation of PSII subunit genes and assembly-related factors under blue light (**Figure 4C**). This concordance between physiological and transcriptomic data suggests that blue light promotes not only the function but also the structural integrity of PSII complexes.

### 4.2 Molecular mechanisms of chlorophyll biosynthesis regulation

Our physiological measurements showed that chlorophyll accumulation in response to light quality was predominantly localized in cotyledons, with hypocotyls containing minimal pigment (**Figures 2A–F**). As chlorophyll biosynthesis occurs primarily in photosynthetically active tissues, the expression changes detected for chlorophyll-related genes in whole-seedling transcriptomes are likely driven by cotyledon-specific signals. The robust differential expression of these genes (**Figure 4**) despite any dilution from hypocotyl tissue further supports that the observed transcriptional changes reliably reflect regulatory events in cotyledons. We therefore analyzed the expression patterns of key chlorophyll biosynthesis genes and their upstream regulators in response to red and blue light.

The chlorophyll biosynthesis pathway demonstrates remarkable spectral sensitivity. Our data identify three critical control points that are differentially regulated by spectral composition: (1) ALA synthesis through *HEMA1*, (2) the Mg-chelatase branching point (*CHLH*/*CHLD* expression), and (3) light-dependent *POR*/*CAO* reactions.

**Figure 5:**
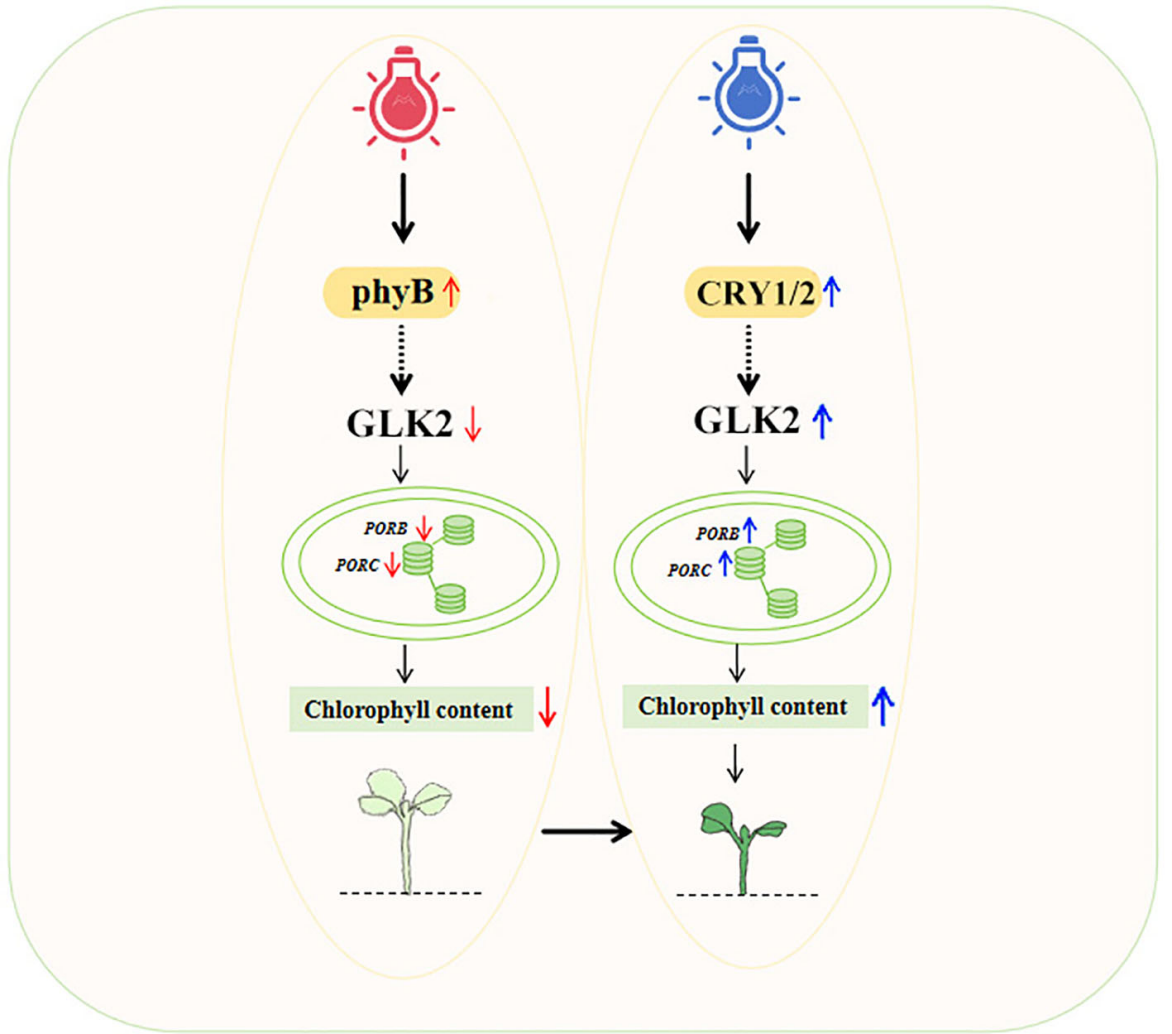
Schematic model of blue- and red- light regulation on microgreens quality. Blue light (Right) is sensed by cryptochrome 1 (CRY1), leading to activation of GLK2 transcription factors. This upregulates photosynthesis and chlorophyll biosynthesis genes, resulting in high pigment content, enhanced photosynthetic efficiency, and increased accumulation of sugars and bioactive compounds. In contrast, red light (left) activates phytochrome B (PhyB), which suppresses chlorophyll biosynthesis through repression of key enzymes (e.g., POR, CAO), leading to reduced pigment accumulation despite promoting morphological growth. These spectral-specific pathways ultimately determine the visual and nutritional quality of microgreens.

Structural insights into PhyB-PIF interactions from cryo-EM studies (Jia et al., 2025) provide a mechanistic basis for our observation of *PIF4/5* downregulation (0.31 fold under red light). In *pif4/5* mutants, the chlorophyll biosynthesis genes were upregulated and chlorophyll accumulated (Shin et al., 2009). The upregulation of *CRY1* and *GLK2* in blue light-treated microgreens corresponds with significant enhancement of chlorophyll biosynthesis and accumulation, demonstrating a light-quality-mediated mechanism for improving nutritional quality in edible microgreens. Previous studies have demonstrated that blue light-activated CRY1 stabilizes ELONGATED HYPOCOTYL 5 (HY5) (Liu et al., 2017, 2025), which binds specifically to G-box elements in promoters of chlorophyll biosynthesis genes *PSY* and *LHCA4* to upregulate their expression (Qu et al., 2014; Zhang et al., 2022). The upregulation of these genes under blue light in our dataset is consistent with this regulatory mechanism. HY5 has been shown to act upstream of *GOLDEN2-LIKE* (*GLK*) by binding to *GLK* promoters to activate their expression, and also interacts with GLK proteins in *Arabidopsis* (Zhang et al., 2024). The coordinated expression patterns observed in our dataset are consistent with this regulatory relationship. In addition, the downregulation of PIF4 can activate GLK regulators by binding the E-box regions of both *GLK1* and *GLK2* promoters (Song et al., 2014). GLK1 and GLK2 are upstream positive regulators of chlorophyll biosynthesis and chloroplast development (Waters et al., 2009). It was reported that PIF4 significantly repressed *GLK2* expression but not *GLK1* (Song et al., 2014). This dual regulatory mechanism, established in previous studies, provides a plausible explanation for the higher chlorophyll content observed under blue light in our study. Based on our transcriptomic data and consistent with established pathways, we propose a working model (**Figure 5**) for light quality regulation of chlorophyll biosynthesis in Chinese kale, which provides testable hypotheses for future functional studies. Recent studies on *Primula veris* have similarly demonstrated that fine-tuning a blue-red LED spectrum can enhance leaf anatomical and physiological traits (Sarropoulou et al., 2025), underscoring the broader applicability of light quality manipulation in controlled environment agriculture. The findings provide a scientific basis for optimizing LED lighting regimens in vertical farming to enhance both the visual quality and nutritional value of microgreens.

We acknowledge several limitations in this study. First, the absence of a white light control limits comparison to standard growth conditions, but this does not affect our core objective of comparing red versus blue light effects. Second, while our transcriptomic data reveal strong correlations, the proposed regulatory model requires validation through functional genetics approaches in future studies.

## 5 Conclusion

This study demonstrates that light quality differentially regulates chlorophyll metabolism in Chinese kale microgreens through wavelength-specific molecular mechanisms. Blue light enhances photosynthetic efficiency and chlorophyll accumulation by upregulating photosystem genes and light-harvesting complexes via CRY-mediated signaling and GLK2 activation, while red light suppresses chlorophyll biosynthesis through PhyB-PIF-mediated repression of POR, CAO, and Mg-chelatase components. These findings provide molecular insights into spectral regulation of plant metabolism and offer practical LED lighting strategies for optimizing microgreen quality in controlled environment agriculture.

## Data Availability

The datasets presented in this study can be found in online repositories. The names of the repository/repositories and accession number(s) can be found in the article/supplementary material.
